# Prevalence of *BRCA1* and *BRCA2* germline variants in an unselected pancreatic cancer patient cohort in Pakistan

**DOI:** 10.1186/s13053-023-00269-x

**Published:** 2023-11-11

**Authors:** Noor Muhammad, Ayesha Azeem, Shumaila Arif, Humaira Naeemi, Iqra Masood, Usman Hassan, Bushra Ijaz, Faisal Hanif, Aamir Ali Syed, Muhammed Aasim Yusuf, Muhammad Usman Rashid

**Affiliations:** 1 Basic Sciences Research, Shaukat Khanum Memorial Cancer Hospital and Research Centre (SKMCH&RC), Lahore, Pakistan; 2 Laboratory of Applied and Functional Genomics, National Center of Excellence in Molecular Biology, University of the Punjab, Lahore, Pakistan; 3 Clinical Research Office, SKMCH&RC, Lahore, Pakistan; 4 Department of Pathology, SKMCH&RC, Lahore, Pakistan; 5 Department of Surgical Oncology, SKMCH&RC, Lahore, Pakistan; 6 Centre for Liver and Biliary Sciences, Bahria International Hospital, Lahore, Pakistan; 7 Department of Internal Medicine, SKMCH&RC, Lahore, Pakistan

**Keywords:** *BRCA1*, *BRCA2*, Germline variants, Pancreatic cancer, Pakistan

## Abstract

**Background:**

*BRCA1* and *BRCA2 (BRCA1/2)* are the most frequently investigated genes among Caucasian pancreatic cancer patients, whereas limited reports are available among Asians. We aimed to investigate the prevalence of *BRCA1/2* germline variants in Pakistani pancreatic cancer patients.

**Methods:**

One hundred and fifty unselected and prospectively enrolled pancreatic cancer patients were comprehensively screened for *BRCA1/2* germline variants using denaturing high-performance liquid chromatography and high-resolution melting analyses, followed by DNA sequencing of the variant fragments. The novel variants were analyzed for their pathogenic effect using *in-silico* tools. Potentially functional variants were further screened in 200 cancer-free controls.

**Results:**

Protein truncating variant was detected in *BRCA2* only, with a prevalence of 0.7% (1/150). A frameshift *BRCA2* variant (p.Asp946Ilefs*14) was identified in a 71-year-old male patient of Pathan ethnicity, with a family history of abdominal cancer. Additionally, we found a novel variant in *BRCA2* (p.Glu2650Gln), two previously reported variants in *BRCA1* (p.Thr293Ser) and *BRCA2* (p.Ile2296Leu) and a recurrent nonsense variant in *BRCA2* (p.Lys3326Ter). These variants were classified as variants of uncertain significance (VUS). It is noteworthy that none of these VUS carriers had a family history of pancreatic or other cancers.

**Conclusions:**

In this first study, *BRCA1/2* pathogenic variant is identified with a low frequency in pancreatic cancer patients from Pakistan. Comprehensive multigene panel testing is recommended in the Pakistani pancreatic cancer patients to enhance genetic understanding in this population.

**Supplementary Information:**

The online version contains supplementary material available at 10.1186/s13053-023-00269-x.

## Background

Pancreatic cancer is the seventh leading cause of cancer associated mortalities worldwide with a very poor prognosis [1]. In Pakistan, 1162 new pancreatic cancer patients were diagnosed in 2020 and the majority of these patients (97.2%) died within the same year [2]. Therefore, it is clinically important to detect individuals at a high risk of developing pancreatic carcinoma early.

Most pancreatic cancer patients are sporadic. About 10–20% are hereditary pancreatic cancer cases due to known genetic risk factors [3], including the pathogenic variants in cancer predisposing genes [4]. Of these genes, *BRCA1* and *BRCA2* (*BRCA1/2*) are the most frequently investigated to assess their contribution to pancreatic cancer risk among Caucasians. *BRCA1/2* pathogenic germline variants have been reported with a frequency of 0.15 and 0.38% in the general population from the UK and USA, respectively [5]. *BRCA1/2* pathogenic variants carriers have approximately 2.5% risk of developing pancreatic cancer by age 80 [6].

The frequency of *BRCA1/2* pathogenic variants in pancreatic cancer patients varies widely across ethnic groups and geographic regions. Previous studies have primarily focused on Caucasians [7–11], with limited reports among Asians, including studies from China [12], Japan [13, 14], and Korea [15]. Studies conducted on unselected pancreatic cancer patients (with > 100 patient cohorts) are summarized in Additional file 1 [see Additional file 1 Table S1]. To our knowledge, there is no study on the contribution of *BRCA1/2* germline pathogenic variants to pancreatic cancer patients in South Asian, including the Pakistani population. Previously, we reported 23.4% prevalence of *BRCA1/2* pathogenic variants among Pakistani high-risk breast and/or ovarian cancer families [16, 17]. Of the identified variants, several population-specific *BRCA1/2* variants have been detected, suggesting founder effects in the Pakistani population [18]. Hence, it is plausible that *BRCA1/2* pathogenic variants might also be associated with pancreatic cancer risk in the Pakistani population. The present study aimed to investigate the prevalence of *BRCA1/2* pathogenic germline variants among pancreatic cancer patients from Pakistan. This study may help identify important genetic biomarkers in Pakistani pancreatic cancer patients. These population-specific genetic biomarkers could serve as specific targets for prospective diagnosis, prognosis, early-stage detection, and personalized medicine.

## Methods

### Study subjects

This study included 150 unrelated, consecutive, and unselected pancreatic cancer patients enrolled at the SKMCH&RC Lahore and SKMCH&RC Peshawar, and Bahria International Hospital, Lahore, between February 2017 and October 2021, as previously described [19]. All patients were histologically confirmed with adenocarcinoma of the pancreas, or ampullary/periampullary region. All pancreatic cancer patients were tested negative for *PALB2* pathogenic variants [19]. The control population included 200 adult individuals (> 18 years) without a personal or family history of any cancer enrolled simultaneously with the cases, as previously described [19]. The study was approved by the Institutional Review Board (IRB) of the Shaukat Khanum Memorial Cancer Hospital and Research Centre (SKMCH&RC), (Approval # IRB-16-14; December 16, 2016). All study participants signed informed written consent prior to the study enrollment. All data were fully anonymized.

Demographics and potential risk factors data (e.g., age at cancer diagnosis for cases, age at enrollment for controls, ethnic background, history of any medical illness, smoking, drinking habits (tea/coffee/alcohol), physical activity and family history of cancer were obtained from all recruited study participants at the time of enrollment using a purposely designed study questionnaire. Clinical and histopathological data of the patients were collected from medical records and pathology reports, respectively between February 2017 and August 2022.

### Molecular analyses

Genomic DNA was extracted from 9 ml to 18 ml of whole blood using the Gentra Puregene kit (Qiagen, Germantown, MD USA), following the manufacturer’s instructions. The complete coding sequences and exon-intron junctions of *BRCA1* (Genbank Accession number NM_007294.3) and *BRCA2* (Genbank Accession number NM_000059.3) were screened in 150 patients. *BRCA1/2* gene scanning was performed by denaturing high-performance liquid chromatography (DHPLC) and high-resolution melting (HRM) analyses using WAVE 4500 DNA Fragment Analysis System (Transgenomics, Omaha, NE USA) and the LightCycler 480-II System (Roche Diagnostics, Indianapolis, IN, USA), respectively. Positive controls for each amplicon were included in every analysis. We established a cost-effective and rapid screening HRM assay and successfully validated it for the screening of coding sequences of *BRCA1* (47%) and *BRCA2* (31%). As positive controls were not identified by HRM analysis for the remaining coding sequences of *BRCA1* (53%) and *BRCA2* (69%), DHPLC was performed [17].

The identified variants were bi-directionally sequenced using an automated 3500 Genetic Analyzer (Applied Biosystems, Foster City CA, USA). Additionally, all patients were screened for two founder large deletions of *BRCA1* exons 1 to 2 and exons 21 to 24, as previously described [20]. The identified pathogenic variant, in silico-predicted likely pathogenic variants, and variants of uncertain significance (VUS) were screened in 200 healthy controls.

### In silico analyses

Novel *BRCA1/2* variants (*n* = 11) and previously reported VUS (*n* = 2) were analyzed using in silico analysis tools. The potential effect of missense variants on protein function was assessed using the default settings of the web tool VarCards, which interprets the pathogenicity of nonsynonymous variants using numerous genomic features and in silico algorithms [21]. The intronic variants were analyzed for their potential effect on splicing using splice prediction tools SpliceSiteFinder-like, MaxEntScan, NNSPLICE, and GeneSplicer using the Alamut Visual Plus v1.2.1 software (SOPHiA GENETICS) in default settings, as previously described [19].

### Variant classification

All identified variants were defined as a novel or previously reported variants by searching the literature and public databases ClinVar (https://www.ncbi.nlm.nih.gov/clinvar/), LOVD (https://databases.lovd.nl/shared/genes/BRCA1), and gnomAD v2.1.1 (https://gnomad.broadinstitute.org/) (by September 2022). All variants were classified as recommended by the American College of Medical Genetics and Genomics and the Association for Molecular Pathology (ACMG/AMP) [22]. The publicly available computational web tools Pathogenicity of Mutation Analyzer (PathoMAN) [23] and InterVar [24] were utilized for the variant classification. These tools use 28 various criteria of ACMG/AMP guidelines and result in preliminary interpretations that can be further reviewed and manually adjusted for final interpretations [23, 24].

## Results

**Fig. 1:**
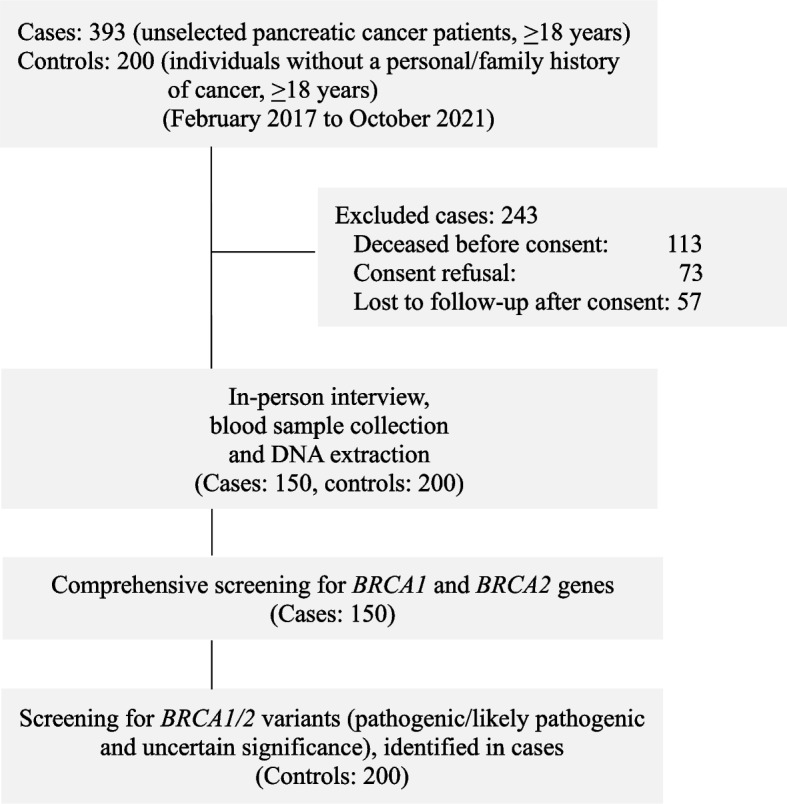
Description of the study participants

In total, 393 pancreatic cancer patients were identified eligible for enrollment in this study. Of these, 243 patients were excluded because they were deceased before consent (*n* = 113), refused to participate in the study (*n* = 73), or lost to follow-up (*n* = 57) (Fig. 1). The remaining 150 unselected pancreatic cancer patients were comprehensively screened for *BRCA1/2* variants using DHPLC and HRM analyses followed by DNA sequencing. The mean age at pancreatic cancer diagnosis was 55.2 years (range 31.2 to 78.4). The majority of the pancreatic cancer patients were male (106/150; 70.7%). Family history of breast, pancreatic, gastrointestinal, or other cancers was reported in 36% (54/150) of the index pancreatic cancer patients. Majority of these patients presented with tumors in the head of the pancreas (108/150; 72%), 2 to 5 cm of tumor size (108/150; 72%), unresectable disease (85/150; 56.7%), and an advanced disease stage III or IV (100/150; 66.7%).

### Spectrum of *BRCA1/2* germline sequence variants

**Fig. 2:**
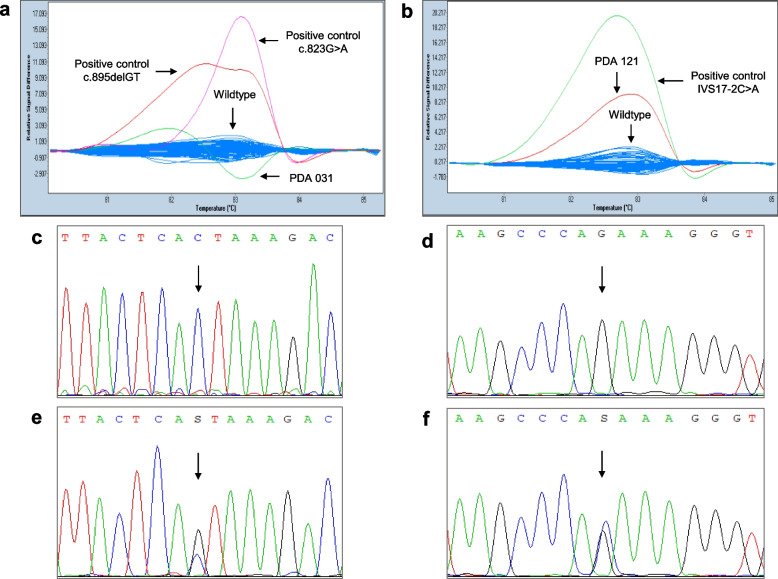
Representative HRM difference plots and DNA sequencing chromatograms of *BRCA1/2* analysis. Normalized and temperature-shifted difference plots of the *BRCA1* **(a)** and *BRCA2* (**b**) showing the discrimination of positive controls and the variant-carriers (PDA 031 and PDA 121) from the wildtypes. DNA sequencing chromatograms of forward DNA strand of wildtypes **(c, d)**, the patient PDA 031 **(e)** showing a *BRCA1* variant (c.878C > G) and the patient PDA 121 (**f**) showing a *BRCA2* variant (c.7948G > C) as indicated by an arrow

In total, 63 different heterozygous variants were identified in *BRCA1* (*n* = 27) and *BRCA2* (*n* = 36) among unselected Pakistani pancreatic cancer patients. Figure 2 displays representative HRM curves and corresponding DNA sequencing chromatograms for *BRCA1* and *BRCA2* analyses.

Of the identified variants, five *BRCA1* variants (one missense and four intronic variants) and seven *BRCA2* variants (five missense, one synonymous, and one intronic variant) were novel and specific to the Pakistani population. The remaining 51 variants in *BRCA1* (*n* = 22) and *BRCA2* (*n* = 29) had been previously reported or described in ClinVar, LOVD, or gnomAD databases. The prevalence and allele frequencies of all identified *BRCA1* and *BRCA2* variants in 150 pancreatic cancer patients are detailed in Table 1 and Table 2, respectively.

**Table 1:** *BRCA1* germline variants identified in Pakistani pancreatic cancer patients

Location	Coding (c.) DNA Sequence^a^ (amino acid change)	SNP ID	Effect	Prevalence, n (%)		Minor allele frequency (%)		Novel or previously reported
				Cases *N* = 150	Controls *N* = 200	Cases	gnomAD, SAS	
*Variant of uncertain significance*								
Exon 11	c.878C > G (p.Thr293Ser)	rs747172803	Missense	1 (0.67)	1 (0.5)	0.333	0.013	ClinVar, LOVD
*Benign/likely benign variants - coding*								
Exon 2	c.36A > G (p.Gln12Gln)	rs763230080	Silent	1 (0.67)	–	0.333	0.029	ClinVar, LOVD
Exon 8	c.536A > G (p.Tyr179Cys)	rs56187033	Missense	1 (0.67)	–	0.333	0.026	ClinVar, LOVD
Exon 11	c.823G > A (p.Gly275Ser)	rs8176153	Missense	2 (1.33)	–	0.667	0.459	ClinVar, LOVD
Exon 11	c.1067A > G (p.Gln356Arg)	rs1799950	Missense	1 (0.67)	–	0.333	1.316	ClinVar, LOVD
Exon 11	c.2077G > A (p.Asp693Asn)	rs4986850	Missense	10 (6.7)	–	3.333	3.536	ClinVar, LOVD
Exon 11	c.2082C > T (p.Ser694Ser)	rs1799949	Silent	34 (22.7)	–	11.33	50.4	ClinVar, LOVD
Exon 11	c.2311 T > C (p.Leu771Leu)	rs16940	Silent	34 (22.7)	–	11.33	50.3	ClinVar, LOVD
Exon 11	c.2521C > T (p.Arg841Trp)	rs1800709	Missense	1 (0.67)	–	0.333	0.183	ClinVar, LOVD
Exon 11	c.2612C > T (p.Pro871Leu)	rs799917	Missense	76 (50.7)	–	25.33	53.16	ClinVar, LOVD
Exon 11	c.2580A > G (p.Thr860Thr)	rs556684572	Silent	1 (0.67)	–	0.333	0.0098	ClinVar, LOVD
Exon 11	c.3113A > G (p.Glu1038Gly)	rs16941	Missense	2 (1.33)	–	0.667	50.35	ClinVar, LOVD
Exon 11	c.3119G > A (p.Ser1040Asn)	rs4986852	Missense	2 (1.33)	–	0.667	0.542	ClinVar, LOVD
Exon 13	c.4308 T > C (p.Ser1436Ser)	rs1060915	Silent	2 (1.33)	–	0.667	50.4	ClinVar, LOVD
Exon 16	c.4837A > G (p.Ser1613Gly)	rs1799966	Missense	8 (5.33)	–	2.667	50.4	ClinVar, LOVD
Exon 16	c.4883 T > C (p.Met1628Thr)	rs4986854	Missense	1 (0.67)	–	0.333	0.042	ClinVar, LOVD
Exon 16	c.4956G > A (p.Met1652Ile)	rs1799967	Missense	4 (2.67)	–	1.333	3.80	ClinVar, LOVD
Exon 20	c.5218G > A (p.Val1740Met)	–	Missense	1 (0.67)	0	0.333	–	Novel
*Benign/likely benign variants - non-coding*								
Intron 7	c.442-34C > T	rs799923	Intronic	31 (20.7)	–	10.33	17.74	ClinVar, LOVD
Intron 8	c.547 + 36A > G	–	Intronic	1 (0.67)	–	0.333	–	Novel
Intron 13	c.4358-61C > G	–	Intronic	1 (0.67)	–	0.333	–	Novel
Intron 14	c.4484 + 14A > G	rs80358022	Intronic	1 (0.67)	–	0.333	0.0653	ClinVar, LOVD
Intron 14	c.4485-63C > G	rs273900734	Intronic	2 (1.33)	–	0.667	0	ClinVar, LOVD
Intron 15	c.4675 + 80C > T	–	Intronic	1 (0.67)	–	0.333	–	Novel
Intron 18	c.5152 + 41 T > C	–	Intronic	1 (0.67)	–	0.333	–	Novel
Intron 18	c.5152 + 66G > A	rs3092994	Intronic	2 (1.33)	–	0.667	0	ClinVar, LOVD
Intron 22	c.5406 + 33A > T	rs80358092	Intronic	1 (0.67)	–	0.333	0.066	ClinVar, LOVD

*gnomAD* Genome Aggregation Database, *LOVD* Leiden Open Variant Database, *SAS* South Asians

^a^Nomenclature follows Human Genome Variation Society (HGVS) (http://www.hgvs.org). Numbering starts at the first A of the first coding ATG (located in exon 2) of NCBI GenBank Accession NM_007294.3

**Table 2:** *BRCA2* germline variants identified in Pakistani pancreatic cancer patients

Location	Coding (c.) DNA Sequence^a^ (amino acid change)	SNP ID	Effect	Prevalence, n (%)		Minor allele frequency (%)		Novel or previously reported
				Cases *n* = 150	Controls *n* = 200	Cases	gnomAD, SAS	
*Pathogenic variant*								
Exon 11	c.2835delA (p.Asp946Ilefs*14)	rs80359356	Frameshift	1 (0.67)	0	0.333	–	ClinVar, LOVD
*Variants of uncertain significance*								
Exon 12	c.6886A > C (p.Ile2296Leu)	rs576279166	Missense	1 (0.67)	0	0.333	0.144	ClinVar, LOVD
Exon 17	c.7948G > C (p.Glu2650Gln)	–	Missense	1 (0.67)	0	0.333	–	Novel
Exon 27	c.9976A > T (p.Lys3326Ter)	rs11571833	Nonsense	2 (1.33)	0	0.667	0.693	ClinVar, LOVD
*Benign/likely benign variants - coding*								
Exon 3	c.198A > G (p.Gln66Gln)	rs28897700	Silent	1 (0.67)	–	0.333	0.777	ClinVar, LOVD
Exon 10	c.865A > C (p.Asn289His)	rs766173	Missense	1 (0.67)	–	0.333	11.56	ClinVar, LOVD
Exon 10	c.1166C > A (p.Pro389Gln)	rs397507263	Missense	1 (0.67)	–	0.333	0.317	ClinVar, LOVD
Exon 10	c.1365A > G (p.Ser455Ser)	rs1801439	Silent	1 (0.67)	–	0.333	11.37	ClinVar, LOVD
Exon 11	c.2229 T > C (p.His743His)	rs1801499	Silent	1 (0.67)	–	0.333	11.42	ClinVar, LOVD
Exon 11	c.2783 T > A (p.Val928Asp)	–	Missense	1 (0.67)	–	0.333	–	Novel
Exon 11	c.2919G > A (p.Ser973Ser)	rs45525041	Silent	1 (0.67)	–	0.333	0.159	ClinVar, LOVD
Exon 11	c.2971A > G (p.Asn991Asp)	rs1799944	Missense	2 (1.33)	–	0.667	11.41	ClinVar, LOVD
Exon 11	c.3396A > G (p.Lys1132Lys)	rs1801406	Silent	2 (1.33)	–	0.667	29.89	ClinVar, LOVD
Exon 11	c.3807 T > C (p.Val1269Val)	rs543304	Silent	1 (0.67)	–	0.333	10.12	ClinVar, LOVD
Exon 11	c.4271C > T (p.Ser1424Phe)	–	Missense	7 (4.67)	–	2.333	–	Novel
Exon 11	c.4258G > T (p.Asp1420Tyr)	rs28897727	Missense	3 (2.0)	–	1.0	0.846	ClinVar, LOVD
Exon 11	c.4928 T > C (p.Val1643Ala)	rs28897731	Missense	2 (1.33)	–	0.667	0.010	ClinVar, LOVD
Exon 11	c.5312G > A (p.Gly1771Asp)	rs80358755	Missense	1 (0.67)	–	0.333	0.016	ClinVar, LOVD
Exon 11	c.5744C > T (p.Thr1915Met)	rs4987117	Missense	4 (2.67)	–	1.333	–	ClinVar, LOVD
Exon 11	c.5986G > A (p.Ala1996Thr)	rs80358833	Missense	1 (0.67)	–	0.333	0.372	ClinVar, LOVD
Exon 12	c.6935A > T (p.Asp2312Val)	rs80358916	Missense	1 (0.67)	–	0.333	0.189	ClinVar, LOVD
Exon 14	c.7242A > G (p.Ser2414Ser)	rs1799955	Silent	50 (33.3)	–	16.66	21.99	ClinVar, LOVD
Exon 14	c.7312G > T (p.Asp2438Tyr)	–	Missense	1 (0.67)	–	0.333	–	Novel
Exon 15	c.7469 T > C (p.Ile2490Thr)	rs11571707	Missense	1 (0.67)	–	0.333	0.1699	ClinVar, LOVD
Exon 17	c.7906 T > C (p.Cys2636Arg)	–	Missense	1 (0.67)	–	0.333	–	Novel
Exon 17	c.7971A > G (p.Lys2657Lys)	–	Silent	1 (0.67)	–	0.333	–	Novel
Exon 19	c.8421G > A (p.Ser2807Ser)	rs371278843	Silent	1 (0.67)	–	0.333	0.2711	ClinVar, LOVD
Exon 22	c.8851G > A (p.Ala2951Thr)	rs11571769	Missense	3 (2.0)	–	1.0	1.417	ClinVar, LOVD
Exon 27	c.10234A > G (p.Ile3412Val)	rs1801426	Missense	2 (1.33)	–	0.667	0.219	ClinVar, LOVD
*Benign/likely benign variants - non-coding*								
3′ UTR	c.1–26G > A	rs1799943	5′ UTR	1 (0.67)	–	0.333	28.47	ClinVar, LOVD
Intron 4	c.653 + 67A > C	rs11571610	Intronic	1 (0.67)	–	0.333	0	ClinVar
Intron 10	c.1910-21A > T	–	Intronic	1 (0.67)	–	0.333	–	Novel
Intron 10	c.1910-74 T > C	rs2320236	Intronic	7 (4.67)	–	2.333	0	ClinVar
Intron 10	c.1910-51G > T	rs11571651	Intronic	29 (19.3)	–	9.667	11.28	ClinVar, LOVD
Intron 14	c.7435 + 53C > T	rs11147489	Intronic	8 (5.33)	–	2.667	0	ClinVar
Intron 21	c.8755-66 T > C	rs4942486	Intronic	2 (1.33)	–	0.667	0	ClinVar

*gnomAD* Genome Aggregation Database, *LOVD* Leiden Open Variant Database, *SAS* South Asians

^a^Nomenclature follows Human Genome Variation Society (HGVS) (http://www.hgvs.org). Numbering starts at the first A of the first coding ATG (located in exon 2) of NCBI GenBank Accession NM_000059.3

The novel variants and previously reported VUS in *BRCA1* (*n* = 6) and *BRCA2* (*n* = 7) were assessed for their putative functional effects on protein or splicing using in silico meta-predictor tools and Alamut splice-site prediction tools, respectively. A novel missense variant in *BRCA2* (p.Glu2650Gln) and two previously reported missense variants in *BRCA1* (p.Thr293Ser) and *BRCA2* (p.Ile2296Leu) were predicted as VUS. The remaining five *BRCA1* variants (p.Val1740Met, c.547 + 36A > G, c.4358-61C > G, c.4675 + 80C > T, and c.5152 + 41 T > C) and five *BRCA2* variants (p.Val928Asp, p.Ser1424Phe, p.Asp2438Tyr, p.Cys2636Arg, and c.1910-21A > T) were predicted as benign/likely benign (Table 3).

**Table 3:** In silico analyses and classification of *BRCA1/2* variants

	VarCards in silico predictions				ACMG-AMP criteria^d^	Classification
**Coding variants**	D/A algorithms^a^	Damaging score^b^	Extreme^c^			
*BRCA1*						
p.Thr293Ser	15/23	0.65	Yes		PM1, PP3, BP1	VUS
p.Val1740Met	18/23	0.78	Yes		PM1, PM2, PP3, BP1, BS3	Likely benign
*BRCA2*						
p.Ile2296Leu	10/23	0.43	No		PM1, PP3, BP1	VUS
p.Glu2650Gln	20/23	0.87	Yes		PM1, PM2, PP3, BP1	VUS
p.Val928Asp	6/23	0.26	No		PM1, PM2, BP1, BP4	Likely benign
p.Ser1424Phe	1/23	0.04	No		PM1, PM2, BP1, BP4	Likely benign
p.Asp2438Tyr	6/23	0.26	No		PM1, PM2, PB1, BP4	Likely benign
p.Cys2636Arg	8/23	0.35	No		PM1, PM2, PB1, BP4	Likely benign
**Non-coding variants**	**Alamut splice-site predictions**^e^				**ACMG-AMP criteria**^d^	**Classification**
	SSF-like	MaxEnt-Scan	NNSPLICE	Gene-Splicer		
*BRCA1*						
c.547 + 36A > G	NE	NE	NE	NE	PM2, BP4	Benign
c.4358-61C > G	NE	NE	NE	NE	PM2, BP4	Benign
c.4675 + 80C > T	NE	NE	NE	NE	PM2, BP4	Benign
c.5152 + 41 T > C	NE	NE	NE	NE	PM2, PP5, BP4	Benign
*BRCA2*						
c.1910-21A > T	NE	NE	NE	NE	PM2, BP4	Benign

*ACMG-AMP* American College of Medical Genetics and Genomics and Association of Molecular Pathology, *NE* no effect, *VUS* variant of uncertain significance

^a^Number of algorithms predicted to be deleterious out of total in silico algorithms

^b^Proportion of algorithms predicted to be deleterious. Damaging score of loss-of-function variants is deemed to be 1

^c^The loss-of-function and damaging (score > 0.5) non-synonymous variants with allele frequency < 0.01% are regard as extreme variants

^d^Predicted by PathoMan and/or InterVar tools with manual adjustment

^e^ > 20% change in score (i.e., a wild-type splice-site score decreases, and/or a cryptic splice-site score increases) is considered as significant

### *BRCA2* pathogenic variant

A *BRCA2* protein-truncating pathogenic variant (p.Asp946Ilefs*14) was detected in an unselected pancreatic cancer patient, accounting for 0.7% (1/150) prevalence in the cohort. This frameshift variant in exon 11 was identified in a 71-year-old male patient (II:2, Fig. 3) of Pathan ethnicity. He presented with grade 3 pancreatic ductal adenocarcinoma with lymphovascular invasion and regional lymph node metastases. His brother (II:3, Fig. 3) was diagnosed with abdominal cancer of unknown primary origin. This variant was absent in 200 unaffected controls and has been previously reported as pathogenic in the ClinVar database.

**Fig. 3:**
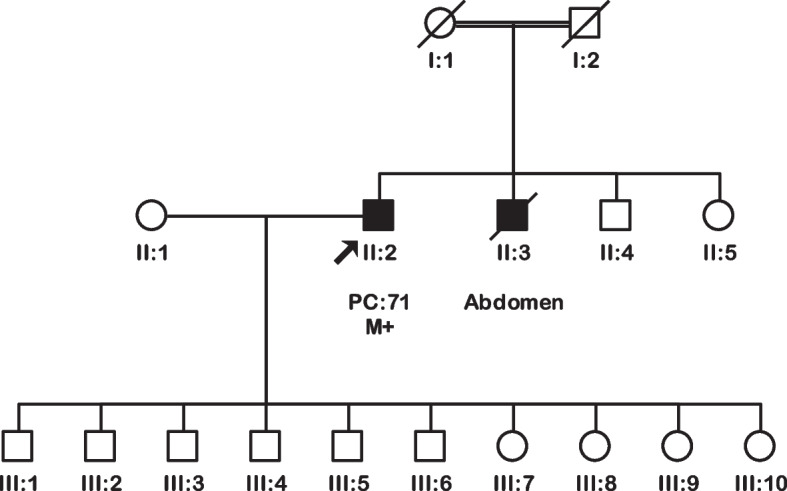
Pedigree of the pancreatic cancer patient carrying a *BRCA2* pathogenic variant. Index patient (PDA 098) carrying the c.2835delA variant. Circles are females, squares are males, and a diagonal slash indicates a deceased individual. Filled symbols show cancer diagnoses. Identification numbers of individuals are below the symbols. The index patient is indicated by an arrow. Double lines show consanguineous marriage. PC: pancreatic cancer. The numbers following the abbreviation indicate the age at cancer diagnosis. M+: variant carrier

### *BRCA1/2* variants of uncertain significance (VUS)

A *BRCA1* missense variant in exon 11, p.Thr293Ser, was detected in a 50-years-old male patient of Punjabi ethnicity. He presented with pancreatic ductal adenocarcinoma with regional lymph node metastases. This variant was also detected in a 59-years-old unaffected female control of Punjabi ethnicity (0.5%;1/200). It has been reported with a low minor allele frequency of 0.013% (4/30,612) among South Asians in gnomAD and further described with conflicting interpretations of pathogenicity in the ClinVar database. It was predicted as pathogenic by 15 of 23 protein function prediction algorithms with VarCards damaging score of 0.65 (Table 3). This variant was predicted to fulfil ACMG/AMP variant interpretation criteria PM1, PP3, and BP1 using InterVar tool. Based on these findings, p.Thr293Ser was classified as VUS.

A *BRCA2* missense variant in exon 12, p.Ile2296Leu, was identified in a 52-years-old male patient of Kashmiri ethnicity. He presented with grade 2 ampullary ductal adenocarcinoma with lymphovascular invasion and regional lymph node metastases. It was not detected in 200 healthy controls. This variant has been reported with a minor allele frequency of 0.14% (44/30,422) among South Asians in gnomAD. It has been reported with conflicting interpretations of pathogenicity in the ClinVar database. It was predicted as pathogenic by 10 of 23 protein function prediction algorithms with VarCards damaging score of 0.43 (Table 3). This variant was predicted to fulfil ACMG/AMP variant interpretation criteria PM1, PP3, and BP1 using the InterVar tool. Based on these findings, p.Ile2296Leu was categorized as VUS.

A *BRCA2* missense variant in exon 17, p.Glu2650Gln, was detected in a 47-years-old female patient of Pathan ethnicity. She had a grade 2 ampullary ductal adenocarcinoma. This variant was not detected in 200 controls. This novel variant has not been reported in literature or databases (gnomAD, ClinVar, and LOVD). It was predicted as pathogenic by 20 of 23 protein function prediction algorithms with VarCards damaging score of 0.87 (Table 3). This variant was predicted to fulfil ACMG/AMP variant interpretation criteria PM1, PM2, PP3, and BP1 using the InterVar tool. Based on these findings, p.Glu2650Gln was classified as VUS.

A recurrent *BRCA2* nonsense variant in exon 27, p.Lys3326Ter, was found in a 46-years-old male patient of Punjabi ethnicity and a 60-years-old female patient of Punjabi ethnicity. They presented with grade 2/3 pancreatic ductal adenocarcinoma with perineural invasion and regional lymph node metastases. This variant was not detected in 200 healthy controls. It has been reported with a minor allele frequency of 0.693% (212/30,594) among South Asians in gnomAD. It has been reported as benign variant in the ClinVar database among breast and/or ovarian cancer patients but no functional evidence is available. Based on these findings, p.Lys3326Ter was classified as VUS for pancreatic cancer risk.

There was no significant difference (*P* = 0.288) observed in the age at pancreatic cancer diagnosis between VUS carriers (mean 50.4 years, range 44.7–60.1 years) and noncarriers (mean 55.2 years, range 31.2–78.4 years). Additionally, none of the VUS carriers reported a family history of pancreatic or any other cancer.

### *BRCA1/2* benign variants

The remaining 26 *BRCA1* variants (12 missense variants, five silent alterations, and nine non-coding variants) and 32 *BRCA2* variants (16 missense variants, nine silent variants, six intronic variants, and one variant in 5’UTR) were classified as benign based on in silico analyses or previously reported elsewhere or described in the ClinVar, LOVD, or gnomAD database (by August 2022) (Table 1, Table 2).

## Discussion

The significance of *BRCA1/2* genetic testing in pancreatic cancer patients is recently recognized among Caucasians, however, limited reports are available among Asians. This is the first comprehensive study investigating the contribution of *BRCA1/2* variants to a cohort of 150 unselected and prospectively registered pancreatic cancer patients from Pakistan. A *BRCA2* pathogenic variant was detected in an unselected pancreatic cancer patient (1/150; 0.7%). Previously reported frequencies of *BRCA1/2* pathogenic variants in unselected pancreatic cancer patients vary across different populations: 1.2% (12/1009) to 3.2% (16/499) in China [12, 25], 3.4% (34/1005) in Japan [13], 1.2% (5/417) to 6.9% (5/72) in Belgium, Czech Republic [26], Greece [27], Italy [28], UK [5], 1.5% (4/274) to 11.3% (17/151) in the USA [8–11], and 2.3% (4/177) to 4.8% (21/437) in Canada [7, 29]. These findings suggest that frequencies of pathogenic variants in genes associated with pancreatic cancer risk vary with ethnicity and geographical distribution of various populations.

A *BRCA2* pathogenic variant (p.Asp946Ilefs*14) was identified in a pancreatic cancer patient with a family history of abdominal cancer. The co-segregation of this variant with abdominal cancer could not be investigated as the brother of the index patient was deceased. It was not detected in the 200 healthy controls. This frameshift variant is considered pathogenic, resulting in a non-functional protein due to premature stop codon or nonsense-mediated decay of the mRNA. It is also described as a pathogenic variant in the ClinVar database. This variant has also been reported in Greek and African-American breast cancer patients [30, 31]. Previously we reported this variant in one early-onset breast cancer patient at age 30 of Pathan ethnicity from Pakistan [17]. In the current study, a patient harbouring this variant presented with pancreatic cancer at age 71 of Pathan ethnicity from Pakistan. Similar findings have previously been reported showing that pathogenic *BRCA2 v*ariant carriers were diagnosed with pancreatic cancer at an older age compared to breast or ovarian cancer patients [32]. These findings suggest a lower penetrance of the pathogenic *BRCA2* variant in pancreatic cancer patients than in breast/ovarian cancer patients.

A novel *BRCA2* missense variant (p.Glu2650Gln) was identified in one pancreatic cancer patient but not detected in 200 healthy controls. This variant is present in a highly conserved oligonucleotide/oligosaccharide-binding (OB) folds within the DNA binding domain of the BRCA2 (amino acids 2482 to 3184) in its C-terminal region. This domain is important for the interaction of BRCA2 with single stranded DNA and then anchoring RAD51 at the site of the damaged DNA for its repair [33]. This variant may reduce the homologous recombination capacity of BRCA2. Neither the literature nor the *BRCA2* variant databases have reported this variant. Twenty out of 23 in silico protein function prediction tools predicted it as likely pathogenic. Based on the ACMG/AMP criteria (PM1, PM2, PP3, BP1), the variant p.Glu2650Gln is classified as a VUS.

A previously reported *BRCA2* missense variant (p.Ile2296Leu) was identified in one pancreatic cancer patient but not in 200 healthy controls. This variant involved a conserved amino acid change. Previously, a minigene functional splicing assay for this variant showed a minor effect on *BRCA2* exon 12 skipping [34]. Previously, it has been reported as VUS in a North Indian breast/ovarian cancer patient [35] and as a benign variant in South African breast/ovarian cancer patients [36]. Ten of 23 in silico protein function prediction tools predicted it as likely pathogenic. The allele frequency of p.Ile2296Leu was 0.14% (44/30,512) among South Asians in the gnomAD database. Conflicting interpretations for this variant have been reported in the ClinVar database. Data regarding the association of this variant with pancreatic cancer risk is lacking. Based on the current evidences, the variant p.Ile2296Leu was classified as VUS for pancreatic cancer risk.

A recurrent *BRCA2* protein truncating variant (p.Lys3326Ter) was detected in two pancreatic cancer patients but not in 200 controls from Pakistan. This variant is localized at the RAD51 binding domain (residues 3265 to 3330) of BRCA2 at its C-terminal region [37]. It creates a premature stop codon, resulting in the loss of terminal 93 amino acids residues necessary for the resolution of stalled replication forks during the DNA repair activity [37]. There are conflicting interpretations related to the pathogenicity of this variant. The minor allele frequency of this variant among South Asians is 0.69% (212/30,594) in gnomAD database. It has been reported as a benign variant in the ClinVar database. However, this variant has been previously reported in association with an increased risk of breast, ovarian, and prostate cancer [38–40]. It has been reported with a frequency of 5.6% (8/144) to 10.3% (3/29) in familial and 2.8% (7/250) in sporadic pancreatic cancer patients from the USA [41, 42]. In a large case-control study including 2935 sporadic pancreatic cancer patients and 5626 controls, mostly of European and Asian origin, p.Lys3326Ter was reported to confer an increased pancreatic cancer risk [43]. This variant has also been reported with increased probability of developing lung cancer among smokers of European ancestry [44]. Whereas, both Pakistani pancreatic cancer patients carrying this variant were non-smokers. This variant deemed involved in pancreatic cancer pathogenesis in association with other environmental genotoxic risk factor(s). Taken together, p.Lys3326Ter is classified as a VUS for pancreatic cancer risk.

No *BRCA1* protein truncating variant was detected in pancreatic cancer patients from Pakistan. This is consistent with the other reports from China [45], Japan [14], the UK [5], the USA [46], and Canada [29]. However, in this study, a previously reported *BRCA1* missense variant (p.Thr293Ser) was identified in one pancreatic cancer patient and one out of 200 unaffected controls. This variant involves a well conserved nucleotide change. It has been previously reported in an ovarian cancer patient of European ancestry in The Cancer Genome Atlas and showed a neutral impact on the BRCA1 function in a cell-based homology-directed recombination assay [47]. It has been documented with conflicting interpretations of pathogenicity in the ClinVar database. Fifteen out of 23 in silico protein function prediction tools predicted it as likely pathogenic. The allele frequency of p.Thr293Ser was low among South Asians as reported in the gnomAD database (4/30,612; 0.013%). Taken together with the previous reports and our interpretations, p.Thr293Ser is classified as VUS.

In the present study, *BRCA1/2* pathogenic variants were not detected in a small group of familial pancreatic cancer patients (*n* = 4), in agreement with other small studies from China (*n* = 9) [12] and Spain (*n* = 43) [48] and a large study from Italy (*n* = 282) [28]. Whereas *BRCA1/2* pathogenic variants have been reported in familial pancreatic cancer patients with a prevalence of 0.8% (5/638) to 7.3% (9/124) in large studies (> 100 patients) from the USA and Canada [9, 49, 50]. These results suggest that the prevalence of pathogenic variants varies by the genetic enrichment of the study subjects, study size, ethnicity, and geographic origin of the study population. Previously, we reported a high prevalence of *BRCA1/2* pathogenic variants (168/718; 23.4%), including 23 *BRCA1* founder mutations in high-risk breast and/or ovarian cancer patients from Pakistan [16–18]. The breast/ovarian cancer patients included in these studies were genetically enriched and primarily selected based on family history or early age at disease presentation. Whereas unselected pancreatic cancer patients were recruited in the current study. This could explain low prevalence of *BRCA1/2* pathogenic variants in our study. Recently, we reported a low prevalence of *PALB2* pathogenic variants (2/150; 1.3%) in a cohort of unselected pancreatic cancer patients from Pakistan [19]. These findings warrant comprehensive multigene panel testing in the Pakistani population.

Our study findings may have potential clinical implications. Tumours of pancreatic cancer patients harbouring *BRCA1/2* heterozygous germline variants show a loss of heterozygosity of the functional wildtype allele and thus are sensitive to platinum agents [3, 4]. Poly (ADP-ribose) polymerase inhibitor is another promising drug for pancreatic cancer patients harbouring *BRCA1/2* pathogenic germline variants [3, 4]. Moreover, predictive genetic testing may be offered to the first-degree relatives of the pathogenic variant carrier, who have a 50% chance of carrying the same pathogenic variant. Females harbouring *BRCA1/2* pathogenic variants are at increased risk of developing breast/ovarian cancer [4]. Likewise, the males carrying the *BRCA1/2* pathogenic variant are at high risk of developing prostate cancer [4]. Several screening and risk-reduction strategies are available for at-risk, unaffected mutation carrier individuals.

The present study stands out for its inclusion of an unselected and prospectively enrolled cohort of pancreatic cancer patients, eliminating case-selection bias and enhancing robustness of our findings. Given the challenging prognosis associated with pancreatic cancer, conducting prospective studies on these patients is inherently difficult, making our study’s approach particularly noteworthy. However, certain limitations were observed in our study. First, we used DHPLC and HRM assays to identify *BRCA1/2* sequencing candidates with a variant detection sensitivity of less than 100%. Consequently, it is conceivable that some pathogenic or likely pathogenic variants might have been missed using these methods. Nonetheless, it is important to note that positive controls for each amplicon were included in all analyses, ensuring the reliability of the results obtained. Second, comprehensive *BRCA1/2* large genomic rearrangements were not assessed, which may underestimate the true variant frequencies reported in this study. Nevertheless, two large genomic rearrangements in *BRCA1* (exons 1–2 deletion and exons 21–24 deletion) previously reported as founder mutations in Pakistan [18] were not identified in the current study. Third, functional assays were not performed for the likely pathogenic variants and the VUS detected in the current study, leaving an avenue for future investigations to explore the functional implications of these variants.

## Conclusions

This is the first comprehensive study investigating the contribution of *BRCA1/2* germline variants to unselected pancreatic cancer patients from Pakistan. Pathogenic variant was exclusively identified in *BRCA2* (0.7%; 1/150). Additionally, five cases were identified as VUS carriers in *BRCA1* (*n* = 1) and *BRCA2* (*n* = 4). These findings suggest a marginal contribution of *BRCA1/2* germline variants to pancreatic cancer risk in the Pakistani population. Genetic testing using multigene panels is recommended for a better understanding of genetic susceptibility in pancreatic cancer patients from Pakistan.

### Supplementary Information

**Additional file 1.**

## Abbreviations

5’UTR: Five prime untranslated region
ACMG: American College of Medical Genetics and Genomics
AMP: Association for Molecular Pathology
*BRCA1*: Breast Cancer gene 1
*BRCA2*: Breast Cancer gene 2
BRCT: BRCA1 C-terminal
BRIP1: BRCA1 interacting protein 1
DHPLC: Denaturing high-performance liquid chromatography
DNA: Deoxyribonucleic acid
gnomAD: Genome Aggregation Database
HRM: High resolution melting
LOVD: Leiden open variant database
*PALB2*: Partner and localizer of BRCA2
PathoMAN: Pathogenicity of Mutation Analyzer
SKMCH&RC: Shaukat Khanum Memorial Cancer Hospital and Research Centre
VUS: Variants of uncertain significance

## References

[1] Rawla P, Sunkara T, Gaduputi V (2019). Epidemiology of pancreatic Cancer: global trends, etiology and risk factors. World. J Oncol..

[2] Global Cancer Observatory 2020. Date accessed November 15, 2022 [https://gco.iarc.fr/today/data/factsheets/populations/586-pakistan-fact-sheets.pdf].

[3] Devico Marciano N, Kroening G, Dayyani F (2022). BRCA-mutated pancreatic Cancer: from discovery to novel treatment paradigms. Cancers..

[4] Kasuga A, Okamoto T, Udagawa S, Mori C, Mie T, Furukawa T, et al. Molecular features and clinical Management of Hereditary Pancreatic Cancer Syndromes and Familial Pancreatic Cancer. Int J Mol Sci. 2022;23(3) 10.3390/ijms23031205. 10.3390/ijms23031205 PMC8835700 35163129

[5] Astiazaran-Symonds E, Kim J (2022). A genome-first approach to estimate prevalence of germline pathogenic variants and risk of pancreatic Cancer in select Cancer susceptibility genes. Cancers (Basel)..

[6] Li S, Silvestri V, Leslie G, Rebbeck TR, Neuhausen SL, Hopper JL (2022). Cancer risks associated with BRCA1 and BRCA2 pathogenic variants. J Clin Oncol..

[7] Smith AL, Wong C, Cuggia A, Borgida A, Holter S, Hall A (2018). Reflex testing for germline BRCA1, BRCA2, PALB2, and ATM mutations in pancreatic Cancer: mutation prevalence and clinical outcomes from two Canadian research registries. JCO Precis Oncol..

[8] Chittenden A, Haraldsdottir S, Ukaegbu C, Underhill-Blazey M, Gaonkar S, Uno H (2021). Implementing systematic genetic counseling and multigene germline testing for individuals with pancreatic Cancer. JCO Oncol Pract..

[9] Hu C, Hart SN, Polley EC, Gnanaolivu R, Shimelis H, Lee KY (2018). Association between inherited germline mutations in Cancer predisposition genes and risk of pancreatic Cancer. JAMA..

[10] Salo-Mullen EE, O'Reilly EM, Kelsen DP, Ashraf AM, Lowery MA, Yu KH (2015). Identification of germline genetic mutations in patients with pancreatic cancer. Cancer..

[11] Shindo K, Yu J, Suenaga M, Fesharakizadeh S, Cho C, Macgregor-Das A (2017). Deleterious germline mutations in patients with apparently sporadic pancreatic adenocarcinoma. J Clin Oncol..

[12] Yin L, Wei J, Lu Z, Huang S, Gao H, Chen J (2022). Prevalence of germline sequence variations among patients with pancreatic Cancer in China. JAMA Netw Open..

[13] Mizukami K, Iwasaki Y, Kawakami E, Hirata M, Kamatani Y, Matsuda K (2020). Genetic characterization of pancreatic cancer patients and prediction of carrier status of germline pathogenic variants in cancer-predisposing genes. EBioMedicine..

[14] Takeuchi S, Doi M, Ikari N, Yamamoto M, Furukawa T (2018). Mutations in BRCA1, BRCA2, and PALB2, and a panel of 50 cancer-associated genes in pancreatic ductal adenocarcinoma. Sci Rep..

[15] Lee K, Yoo C, Kim KP, Park KJ, Chang HM, Kim TW (2018). Germline BRCA mutations in Asian patients with pancreatic adenocarcinoma: a prospective study evaluating risk category for genetic testing. Investig New Drugs..

[16] Rashid MU, Zaidi A, Torres D, Sultan F, Benner A, Naqvi B (2006). Prevalence of BRCA1 and BRCA2 mutations in Pakistani breast and ovarian cancer patients. Int J Cancer..

[17] Rashid MU, Muhammad N, Naeemi H, Khan FA, Hassan M, Faisal S (2019). Spectrum and prevalence of BRCA1/2 germline mutations in Pakistani breast cancer patients: results from a large comprehensive study. Hered Cancer Clin Pract..

[18] Rashid MU, Muhammad N, Naeemi H, Shehzad U, Hamann U (2022). Chasing the origin of 23 recurrent BRCA1 mutations in Pakistani breast and ovarian cancer patients. Int J Cancer..

[19] Muhammad N, Sadaqat R, Naeemi H, Masood I, Hassan U, Ijaz B (2022). Contribution of germline PALB2 variants to an unselected and prospectively registered pancreatic cancer patient cohort in Pakistan. HPB (Oxford)..

[20] Rashid MU, Muhammad N, Amin A, Loya A, Hamann U (2017). Contribution of BRCA1 large genomic rearrangements to early-onset and familial breast/ovarian cancer in Pakistan. Breast Cancer Res Treat..

[21] Li J, Shi L, Zhang K, Zhang Y, Hu S, Zhao T (2018). VarCards: an integrated genetic and clinical database for coding variants in the human genome. Nucleic Acids Res..

[22] Richards S, Aziz N, Bale S, Bick D, Das S, Gastier-Foster J (2015). Standards and guidelines for the interpretation of sequence variants: a joint consensus recommendation of the American College of Medical Genetics and Genomics and the Association for Molecular Pathology. Genet Med..

[23] Joseph V, Ravichandran V, Offit K (2017). Pathogenicity of mutation analyzer (PathoMAN): a fast automation of germline genomic variant curation in clinical sequencing. J Clin Oncol..

[24] Li Q, Wang K (2017). InterVar: clinical interpretation of genetic variants by the 2015 ACMG-AMP guidelines. Am J Hum Genet..

[25] Zhao Z, Li X (2023). Pathogenic genomic alterations in Chinese pancreatic cancer patients and their therapeutical implications. Cancer Med..

[26] Wieme G, Kral J, Rosseel T (2021). Prevalence of germline pathogenic variants in Cancer predisposing genes in Czech and Belgian pancreatic Cancer patients. Cancers..

[27] Fountzilas E, Eliades A (2021). Clinical significance of germline Cancer predisposing variants in unselected patients with pancreatic adenocarcinoma. Cancers (Basel)..

[28] Peretti U, Cavaliere A, Niger M, Tortora G, Di Marco MC, Rodriquenz MG (2021). Germinal BRCA1-2 pathogenic variants (gBRCA1-2pv) and pancreatic cancer: epidemiology of an Italian patient cohort. ESMO Open..

[29] Cremin C, Lee MK (2020). Burden of hereditary cancer susceptibility in unselected patients with pancreatic ductal adenocarcinoma referred for germline screening. Cancer Med..

[30] Armakolas A, Ladopoulou A, Konstantopoulou I, Pararas B, Gomatos IP, Kataki A (2002). BRCA2 gene mutations in Greek patients with familial breast cancer. Hum Mutat..

[31] Purrington KS, Raychaudhuri S (2020). Heritable susceptibility to breast Cancer among African-American women in the Detroit research on Cancer survivors study. Cancer Epidemiol Biomark Prev..

[32] Cavanagh H, Rogers KM (2015). The role of BRCA1 and BRCA2 mutations in prostate, pancreatic and stomach cancers. Hered Cancer Clin Pract..

[33] Fradet-Turcotte A, Sitz J, Grapton D, Orthwein A (2016). BRCA2 functions: from DNA repair to replication fork stabilization. Endocr Relat Cancer..

[34] Meulemans L, Mesman RLS (2020). Skipping nonsense to maintain function: the paradigm of BRCA2 exon 12. Cancer Res..

[35] Mehta A, Vasudevan S, Sharma SK, Kumar D, Panigrahi M, Suryavanshi M (2018). Germline BRCA1 and BRCA2 deleterious mutations and variants of unknown clinical significance associated with breast/ovarian cancer: a report from North India. Cancer Manag Res..

[36] Van der Merwe NC, Combrink HM, Ntaita KS, Oosthuizen J (2022). Prevalence of clinically relevant germline BRCA variants in a large unselected south African breast and ovarian Cancer cohort: a public sector experience. Front Genet..

[37] Baughan S, Tainsky MA (2021). K3326X and other C-terminal BRCA2 variants implicated in hereditary Cancer syndromes: a review. Cancers (Basel)..

[38] Meeks HD, Song H, Michailidou K, Bolla MK, Dennis J, Wang Q, et al. BRCA2 polymorphic stop codon K3326X and the risk of breast, prostate, and ovarian cancers. J Natl Cancer Inst. 2016;108(2) 10.1093/jnci/djv315. 10.1093/jnci/djv315 PMC4907358 26586665

[39] Stafford JL, Dyson G, Levin NK, Chaudhry S, Rosati R, Kalpage H (2017). Reanalysis of BRCA1/2 negative high risk ovarian cancer patients reveals novel germline risk loci and insights into missing heritability. PLoS One..

[40] Thompson ER, Gorringe KL, Rowley SM, Li N, McInerny S, Wong-Brown MW (2015). Reevaluation of the BRCA2 truncating allele c.9976A > T (p.Lys3326Ter) in a familial breast cancer context. Sci Rep..

[41] Martin ST, Matsubayashi H, Rogers CD, Philips J, Couch FJ, Brune K (2005). Increased prevalence of the BRCA2 polymorphic stop codon K3326X among individuals with familial pancreatic cancer. Oncogene..

[42] Murphy KM, Brune KA, Griffin C, Sollenberger JE, Petersen GM, Bansal R (2002). Evaluation of candidate genes MAP2K4, MADH4, ACVR1B, and BRCA2 in familial pancreatic cancer: deleterious BRCA2 mutations in 17%. Cancer Res..

[43] Obazee O, Archibugi L, Andriulli A, Soucek P, Małecka-Panas E, Ivanauskas A (2019). Germline BRCA2 K3326X and CHEK2 I157T mutations increase risk for sporadic pancreatic ductal adenocarcinoma. Int J Cancer..

[44] Wang Y, McKay JD, Rafnar T, Wang Z, Timofeeva MN, Broderick P (2014). Rare variants of large effect in BRCA2 and CHEK2 affect risk of lung cancer. Nat Genet..

[45] Jiang H, Huang F (2023). Germline mutations in homologous recombination repair genes among Chinese pancreatic ductal adenocarcinoma patients detected using next-generation sequencing. Mol Genet Genomic Med..

[46] Koptiuch C, Espinel WF, Kohlmann WK, Zhao J, Kaphingst KA. Implications of multigene panel testing on psychosocial outcomes: a comparison of patients with pancreatic and breast or ovarian Cancer. JCO Precis Oncol. 2021;5 10.1200/po.20.00199. 10.1200/PO.20.00199 PMC8232274 34250392

[47] Lu C, Xie M, Wendl MC, Wang J, McLellan MD, Leiserson MD (2015). Patterns and functional implications of rare germline variants across 12 cancer types. Nat Commun..

[48] Earl J, Galindo-Pumariño C, Encinas J, Barreto E, Castillo ME, Pachón V (2020). A comprehensive analysis of candidate genes in familial pancreatic cancer families reveals a high frequency of potentially pathogenic germline variants. EBioMedicine..

[49] Dudley B, Karloski E, Monzon FA, Singhi AD (2018). Germline mutation prevalence in individuals with pancreatic cancer and a history of previous malignancy. Cancer..

[50] Roberts NJ, Norris AL, Petersen GM, Bondy ML, Brand R, Gallinger S (2016). Whole genome sequencing defines the genetic heterogeneity of familial pancreatic Cancer. Cancer Discov..

