# Supplementary material for: Prevalence of BRCA1 and BRCA2 germline variants in an unselected pancreatic cancer patient cohort in Pakistan
Source: Hered Cancer Clin Pract. 2023 Nov 11;21:22. doi: 10.1186/s13053-023-00269-x (PMC10640758; doi:10.1186/s13053-023-00269-x)
Supplement: Supplementary file 1 — Additional file 1. [file 13053_2023_269_MOESM1_ESM.docx]

**Additional file 1**

**Table S1** Prevalence of *BRCA1/2* germline pathogenic variants in unselected pancreatic cancer patients

| **Country** | **No of Cases** | **Pathogenic variants,**  **n (%)** | | **Methodology** | **References** |
| --- | --- | --- | --- | --- | --- |
|  |  | ***BRCA1*** | ***BRCA2*** |  |  |
| **Asia** | | |  |  |  |
| China | 1009 | 3 (0.3) | 9 (0.9) | NGS (59 genes) | [[1](#_ENREF_1)] |
| China | 499 | 3 (0.6) | 13 (2.6) | NGS (831 genes) | [[2](#_ENREF_2)] |
| China | 256 | 0 | 4 (1.6) | NGS (21 genes) | [[3](#_ENREF_3)] |
| China | 195 | 0 | 5 (2.6) | NGS (150-417 genes) | [[4](#_ENREF_4)] |
| Japan | 1005 | 9 (0.9) | 25 (2.5) | NGS (27 genes) | [[5](#_ENREF_5)] |
| Pakistan | 150 | 0 | 1 (0.7) | DHPLC, HRM, Sanger sequencing | Current study |
| **Europe** |  |  |  |  |  |
| Czech Republic | 226 | 3 (1.3) | 9 (3.4) | NGS (53 genes) | [[6](#_ENREF_6)] |
| Greece | 549 | 2 (0.4) | 6 (1.1) | NGS (62 genes) | [[7](#_ENREF_7)] |
| Italy | 939 | 12 (1.3) | 64 (6.8) | Sanger sequencing, MLPA | [[8](#_ENREF_8)] |
| UK | 417 | 0 | 5 (1.2) | NGS (whole exome) | [[9](#_ENREF_9)] |
| **North/South America** | |  |  |  |  |
| Canada | 437 | 4 (0.9) | 17 (3.9) | NGS (whole exome, whole genome) | [[10](#_ENREF_10)] |
| Canada | 386 | 2 (0.5) | 16 (4.1) | BFDS, MLPA | [[11](#_ENREF_11)] |
| Canada | 306 | 3 (1.0) | 11 (3.6) | Sanger sequencing, MLPA | [[12](#_ENREF_12)] |
| Canada | 177 | 0 | 4 (2.3) | NGS (30 genes) | [[13](#_ENREF_13)] |
| USA | 3030 | 18 (0.6) | 59 (1.9) | NGS (21 genes) | [[14](#_ENREF_14)] |
| USA | 854 | 3 (0.4) | 12 (1.4) | NGS (32 genes) | [[15](#_ENREF_15)] |
| USA | 592 | 2 (0.34) | 9 (1.52) | NGS (whole exome) | [[9](#_ENREF_9)] |
| USA | 289 | 3 (1.0) | 4 (1.4) | NGS (24 genes) | [[16](#_ENREF_16)] |
| USA | 266 | 4 (1.5) | 7 (2.6) | NGS (multigene) | [[17](#_ENREF_17)] |
| USA | 250 | 1 (0.4) | 9 (3.6) | NGS (83 genes) | [[18](#_ENREF_18)] |
| USA | 151 | 4 (2.6) | 13 (8.6) | Sanger sequencing, MLPA | [[19](#_ENREF_19)] |
| USA | 133 | 2 (1.5) | 2 (1.5) | NGS (263 genes) | [[20](#_ENREF_20)] |
| **Multiethnic** | 2206 | 48 (2.2) | 112 (5.1) | BRCAnalysis | [[21](#_ENREF_21)] |
| **Meta-analysis** | 14658 | 132 (0.9) | 557 (3.8) | Meta-analysis | [[22](#_ENREF_22)] |

*BFDS* bead-based fluorescence DNA sequencing, *DHPLC* denaturing high-performance liquid chromatography, *HDA* heteroduplex analysis, *HRM* high-resolution melting, *MLPA* multiplex ligation-dependent probe amplification, *NGS* next-generation sequencing.

**References**

1. Yin L, Wei J, Lu Z, Huang S, Gao H, Chen J, et al. Prevalence of Germline Sequence Variations Among Patients With Pancreatic Cancer in China. JAMA Netw Open. 2022;5(2):e2148721. https://doi.org/10.1001/jamanetworkopen.2021.48721.

2. Zhao Z, Li X. Pathogenic genomic alterations in Chinese pancreatic cancer patients and their therapeutical implications. Cancer Medicine. 2023;12(10):11672-11685. https://doi.org/10.1002/cam4.5871.

3. Jiang H, Huang F. Germline mutations in homologous recombination repair genes among Chinese pancreatic ductal adenocarcinoma patients detected using next-generation sequencing. Mol Genet Genomic Med. 2023;11(7):e2170. https://doi.org/10.1002/mgg3.2170.

4. Shui L, Li X, Peng Y, Tian J, Li S, He D, et al. The germline/somatic DNA damage repair gene mutations modulate the therapeutic response in Chinese patients with advanced pancreatic ductal adenocarcinoma. J Transl Med. 2021;19(1):301. https://doi.org/10.1186/s12967-021-02972-6.

5. Mizukami K, Iwasaki Y, Kawakami E, Hirata M, Kamatani Y, Matsuda K, et al. Genetic characterization of pancreatic cancer patients and prediction of carrier status of germline pathogenic variants in cancer-predisposing genes. EBioMedicine. 2020;60:103033. https://doi.org/10.1016/j.ebiom.2020.103033.

6. Wieme G, Kral J, Rosseel T. Prevalence of Germline Pathogenic Variants in Cancer Predisposing Genes in Czech and Belgian Pancreatic Cancer Patients. Cancers 2021;13(17). https://doi.org/10.3390/cancers13174430.

7. Fountzilas E, Eliades A. Clinical Significance of Germline Cancer Predisposing Variants in Unselected Patients with Pancreatic Adenocarcinoma. Cancers (Basel). 2021;13(2):198. https://doi.org/10.3390/cancers13020198.

8. Peretti U, Cavaliere A, Niger M, Tortora G, Di Marco MC, Rodriquenz MG, et al. Germinal BRCA1-2 pathogenic variants (gBRCA1-2pv) and pancreatic cancer: epidemiology of an Italian patient cohort. ESMO Open. 2021;6(1):100032. https://doi.org/10.1016/j.esmoop.2020.100032.

9. Astiazaran-Symonds E, Kim J. A Genome-First Approach to Estimate Prevalence of Germline Pathogenic Variants and Risk of Pancreatic Cancer in Select Cancer Susceptibility Genes. Cancers (Basel). 2022;14(13). https://doi.org/10.3390/cancers14133257.

10. Grant RC, Holter S, Borgida A, Dhani NC, Hedley DW, Knox JJ, et al. Comparison of Practice Guidelines, BRCAPRO, and Genetic Counselor Estimates to Identify Germline BRCA1 and BRCA2 Mutations in Pancreatic Cancer. J Genet Couns. 2018;27(4):988-995. https://doi.org/10.1007/s10897-018-0212-1.

11. Smith AL, Wong C, Cuggia A, Borgida A, Holter S, Hall A, et al. Reflex Testing for Germline BRCA1, BRCA2, PALB2, and ATM Mutations in Pancreatic Cancer: Mutation Prevalence and Clinical Outcomes From Two Canadian Research Registries. JCO Precis Oncol. 2018;2:1-16. https://doi.org/10.1200/po.17.00098.

12. Holter S, Borgida A, Dodd A, Grant R, Semotiuk K, Hedley D, et al. Germline BRCA Mutations in a Large Clinic-Based Cohort of Patients With Pancreatic Adenocarcinoma. J Clin Oncol. 2015;33(28):3124-3129. https://doi.org/10.1200/jco.2014.59.7401.

13. Cremin C, Lee MK. Burden of hereditary cancer susceptibility in unselected patients with pancreatic ductal adenocarcinoma referred for germline screening. Cancer Med. 2020;9(11):4004-4013. https://doi.org/10.1002/cam4.2973.

14. Hu C, Hart SN, Polley EC, Gnanaolivu R, Shimelis H, Lee KY, et al. Association Between Inherited Germline Mutations in Cancer Predisposition Genes and Risk of Pancreatic Cancer. Jama. 2018;319(23):2401-2409. https://doi.org/10.1001/jama.2018.6228.

15. Shindo K, Yu J, Suenaga M, Fesharakizadeh S, Cho C, Macgregor-Das A, et al. Deleterious Germline Mutations in Patients With Apparently Sporadic Pancreatic Adenocarcinoma. J Clin Oncol. 2017;35(30):3382-3390. https://doi.org/10.1200/jco.2017.72.3502.

16. Yurgelun MB, Chittenden AB, Morales-Oyarvide V, Rubinson DA, Dunne RF, Kozak MM, et al. Germline cancer susceptibility gene variants, somatic second hits, and survival outcomes in patients with resected pancreatic cancer. Genet Med. 2019;21(1):213-223. https://doi.org/10.1038/s41436-018-0009-5.

17. Chittenden A, Haraldsdottir S, Ukaegbu C, Underhill-Blazey M, Gaonkar S, Uno H, et al. Implementing Systematic Genetic Counseling and Multigene Germline Testing for Individuals With Pancreatic Cancer. JCO Oncol Pract. 2021;17(2):e236-e247. https://doi.org/10.1200/op.20.00678.

18. Uson PLS, Jr., Samadder NJ, Riegert-Johnson D, Boardman L, Borad MJ, Ahn D, et al. Clinical Impact of Pathogenic Germline Variants in Pancreatic Cancer: Results From a Multicenter, Prospective, Universal Genetic Testing Study. Clin Transl Gastroenterol. 2021;12(10):e00414. https://doi.org/10.14309/ctg.0000000000000414.

19. Salo-Mullen EE, O'Reilly EM, Kelsen DP, Ashraf AM, Lowery MA, Yu KH, et al. Identification of germline genetic mutations in patients with pancreatic cancer. Cancer. 2015;121(24):4382-4388. https://doi.org/10.1002/cncr.29664.

20. Goldstein JB, Zhao L, Wang X, Ghelman Y, Overman MJ. Germline DNA Sequencing Reveals Novel Mutations Predictive of Overall Survival in a Cohort of Patients with Pancreatic Cancer. Clin Cancer Res. 2020;26(6):1385-1394. https://doi.org/10.1158/1078-0432.ccr-19-0224.

21. Golan T, Kindler HL, Park JO, Reni M, Macarulla T, Hammel P, et al. Geographic and Ethnic Heterogeneity of Germline BRCA1 or BRCA2 Mutation Prevalence Among Patients With Metastatic Pancreatic Cancer Screened for Entry Into the POLO Trial. J Clin Oncol. 2020;38(13):1442-1454. https://doi.org/10.1200/jco.19.01890.

22. Casolino R, Paiella S, Azzolina D, Beer PA. Homologous Recombination Deficiency in Pancreatic Cancer: A Systematic Review and Prevalence Meta-Analysis. J Clin Oncol. 2021;39(23):2617-2631. https://doi.org/10.1200/jco.20.03238.
